# Treatment implications of posterior fossa ependymoma subgroups

**DOI:** 10.1186/s40880-016-0155-6

**Published:** 2016-11-15

**Authors:** Vijay Ramaswamy, Michael D. Taylor

**Affiliations:** 1 Division of Haematology/Oncology, Hospital for Sick Children, 555 University Avenue, Toronto, ON M5G 1X8 Canada; 2 Division of Neurosurgery, Hospital for Sick Children, 555 University Avenue, Toronto, ON M5G 1X8 Canada

**Keywords:** Posterior fossa ependymoma, Genomics, Radiation, PFA

## Abstract

Posterior fossa ependymoma comprises two distinct molecular entities, ependymoma_posterior fossa A (EPN_PFA) and ependymoma_posterior fossa B (EPN_PFB), with differentiable gene expression profiles. As yet, the response of the two entities to treatment is unclear. To determine the relationship between the two molecular subgroups of posterior fossa ependymoma and treatment, we studied a cohort of 820 patients with molecularly profiled, clinically annotated posterior fossa ependymomas. We found that the strongest predictor of poor outcome in patients with posterior fossa ependymoma across the entire age spectrum was molecular subgroup EPN_PFA, which was recently reported in the paper entitled “Therapeutic impact of cytoreductive surgery and irradiation of posterior fossa ependymoma in the molecular era: a retrospective multicohort analysis” in the *Journal of Clinical Oncology*. Patients with incompletely resected EPN_PFA tumors had a very poor outcome despite receiving adjuvant radiation therapy, whereas a substantial proportion of patients with EPN_PFB tumors can be cured with surgery alone.

Ependymoma is the third most common pediatric brain tumor and can occur anywhere along the neuroaxis, most commonly in the posterior fossa, supratentorium, and spinal cord. One of the biggest challenges in pediatric neuro-oncology is ependymoma of the posterior fossa. Posterior fossa ependymoma has a bimodal age distribution, with peak ages at 5 and 35 years of age [1]. Historically, all ependymomas were considered a single entity; however, over the past 10 years, it has become clear that ependymomas from each of the three anatomical compartments (the posterior fossa, supratentorial, and spinal) are distinct biological entities [2–4]. Recently, our group and others showed that beyond the biological and clinical heterogeneity that are apparent within the anatomical compartments, there is clear heterogeneity within posterior fossa ependymoma [1, 3–13]. We showed that posterior fossa ependymoma comprises two distinct molecular entities called ependymoma_posterior fossa A (EPN_PFA) and ependymoma_posterior fossa B (EPN_PFB). EPN_PFA tumors occur in younger children, result in a poor outcome, and, with the exception of a gain of chromosome 1q in 20% of patients, have a balanced copy number profile and no recurrent somatic nucleotide variants [3, 14]. EPN_PFA tumors have a distinct DNA methylation profile with hypermethylated loci converging on targets of polycomb repressor complex 2 (PRC2) [5]. EPN_PFB tumors occur more frequently in old children and adults, result in a more favorable outcome (compared with EPN_PFA tumors), and have many broad copy number changes with no recurrent somatic nucleotide variants [14].

Current therapy for posterior fossa ependymoma in children older than 1 year consists of maximal safe surgical resection followed by 54–59 Gy of external beam irradiation to the tumor bed [15]. Although several trials of chemotherapy for posterior fossa ependymoma have been conducted in the past 20 years, no chemotherapy regimen has shown clear activity against this disease; however, a randomized trial of maintenance chemotherapy by the Children’s Oncology Group (ACNS0831) is nearing accrual [16, 17]. Although no clear clinical risk stratification exists for posterior fossa ependymoma, incomplete surgical resection has been shown to predict poor prognosis [1]. Currently, a histologic classification divides ependymoma into grade II and grade III; however, this classification has been shown to have very poor interobserver reliability, resulting in limited to no clinical utility [18]. As such, a new approach to the treatment and classification of posterior fossa ependymoma is urgently needed.

Recently, as reported in our *Journal of Clinical Oncology* article entitled “Therapeutic impact of cytoreductive surgery and irradiation of posterior fossa ependymoma in the molecular era: a retrospective multicohort analysis” [19], we studied 820 patients with clinically annotated posterior fossa ependymoma across four independent, non-overlapping cohorts, including three retrospective cohorts and one prospective cohort. These 820 patients were subgrouped using the recently published methylation-based classification of ependymoma [14]. Using a multivariable model incorporating age, extent of surgical resection, postoperative first-line radiotherapy, adjuvant chemotherapy, and molecular subgroup, we found that the strongest predictor of poor outcome was EPN_PFA subgroup, with a hazard ratio of 2.14 (95% confidence interval [CI] 1.31–3.49) for progression and 4.3 (95% CI 1.88–9.87) for death. Incomplete surgical resection and no adjuvant first-line radiotherapy were also associated with a significantly poor outcome. When we performed this analysis in each of the four individual cohorts, we found the same result, confirming the pooled analysis.

As previously described, patients with EPN_PFA tumors were significantly younger than patients with EPN_PFB tumors (no patient under 5 years of age had an EPN_PFB tumor), and more older patients were identified with EPN_PFB tumors [3]. To determine if EPN_PFA tumors are higher risk factor for relapse/progression across the age spectrum, first we plotted the hazard ratio of EPN_PFA vs. EPN_PFB across the age spectrum and found EPN_PFA to be a high risk factor for relapse/progression across all ages. Then, looking at EPN_PFA and EPN_PFB cases individually, we divided the EPN_PFA patients into above or equal to and below 10 years of age and the EPN_PFB patients into above or equal to and below 18 years of age. We found the prognostic implications of molecular subgroup to be independent of age at diagnosis.

Next, we investigated whether the previously observed therapeutic value of surgical cytoreduction holds true within each of the two subgroups. To address this, within each subgroup we compared progression-free survival rates and overall survival rates between patients who underwent gross total resection and those who were treated with subtotal resection (>5 mm residual disease postoperatively). When comparing gross totally resected cases to subtotally resected cases, we found that subtotal resection of EPN_PFA tumors was highly predictive of very poor outcome. We observed this profound difference in outcomes across all four cohorts when stratified by extent of surgical resection. Interestingly, sex had a significant influence: women with a gross total resection had a 5-year progression-free survival rate of 65.2% (95% CI 58.1%–73.2%), whereas men with a gross total resection had a 5-year progression-free survival rate of 45.5% (95% CI 39.3%–52.7%). Both men and women who received a subtotal resection had a very poor outcome. By examining the value of postoperative first-line external beam irradiation for EPN_PFA tumors, we found that irradiation in the setting of gross total resection resulted in a significant benefit; however, this benefit was much less profound in the setting of subtotal resection. We found that, across the entire cohort, adjuvant first-line chemotherapy was of little to no benefit. As such, we concluded that, despite the addition of external beam irradiation, patients with subtotally resected EPN_PFA tumors are at very high risk for relapse/progression and should be urgently prioritized to receive novel therapies.

Additionally, we investigated the role of surgery and irradiation in EPN_PFB tumors. Extent of resection was an important prognostic marker: patients with gross totally resected EPN_PFB tumors had a 10-year overall survival rate of over 90%. However, we observed a significant difference when comparing progression-free survival with overall survival, suggesting that EPN_PFB patients can successfully receive salvage therapy at the time of first relapse. Strikingly, we found that a significant proportion of patients with EPN_PFB tumors could be cured without external beam irradiation and with complete resection alone; and if they did experience a recurrence, they could be salvaged with external beam irradiation. This suggests that, after receiving irradiation, patients with gross totally resected EPN_PFB tumors have an overall survival of over 90%. Furthermore, our observation that some patients could be cured without irradiation indicates that a carefully controlled study of observation alone compared with radiation treatment after complete surgical resection needs to be done in patients with EPN_PFB tumors.

Finally, to predict both progression-free survival rate and overall survival rate, we created a risk-stratification nomogram of posterior fossa ependymoma that incorporated molecular subgroup, first-line irradiation, and extent of resection. This nomogram enables a far more robust risk-stratification analysis to help guide the next generation of clinical trials. Our results are in stark contrast to studies of medulloblastoma, in which the value of extent of resection was significantly dampened when accounting for molecular subgroup [20]. We suggest that complete surgical resection should be attempted in all patients with posterior fossa ependymoma, including those with high-risk EPN_PFA tumors, and that focal external beam irradiation to the tumor bed should be the postoperative standard of care.

## Conclusion and future directions

We assembled the largest-ever cohort of patients with posterior fossa ependymoma, and we identified a group of patients who were at very high risk for relapse/progression and death. Patients with subtotally resected EPN_PFA tumors have a very poor prognosis, and the key clinical goal is to develop therapies that result in improved outcomes. In many patients, complete resection results in significant neurological morbidity because of brainstem involvement, particularly in patients with EPN_PFA tumors. Indeed, in many instances, because of cranial nerve invasion and intricate brainstem invasion, complete resection is impossible. Our results set the stage for additional studies to evaluate appropriate treatment protocols for these patients. Our results suggest a limited role for adjuvant radiotherapy (and chemotherapy) in patients who have incomplete tumor resection. Clinical trials for upfront novel therapeutics are urgently needed for these patients. Several candidate therapeutic approaches have been proposed, including epigenetic modifiers such as polycomb repressor complex 2 (PRC2) inhibition, DNA methylation inhibitors, and histone deacetylase (HDAC) inhibitors, either alone or in combination. Indeed, regardless of the therapeutic approach, both new therapeutic approaches and rapid translation of preclinical findings are needed for children with subtotally resected EPN_PFA tumors. Considering the very poor outcomes of this group with the current standard of care, novel therapeutics should be prioritized upfront, rather than at the time of progression or recurrence, to rapidly determine activity against the residual disease.

Our data suggest that a significant proportion of adolescents and adults with EPN_PFB tumors can be cured without external beam irradiation. Given the significant negative effects that external beam irradiation has on learning and memory, a carefully conducted clinical trial of observation alone for completely resected EPN_PFB tumors should be considered. This will have to be a multinational effort, likely requiring close cooperation between adult and pediatric cooperative groups, since most adults with ependymoma are not treated on open protocols.

The current generation of clinical trials for ependymoma, which were designed over a decade ago, still consider all ependymomas to be one entity and do not incorporate any molecular stratification. Considering the profound clinical and therapeutic differences observed in our study, molecularly informed clinical trials for posterior fossa ependymomas are urgently needed to prioritize novel therapies for patients at high risk, while minimizing the adverse effects of treatment for those patients at low risk.
